# Salmagundi

**Published:** 1888-10

**Authors:** 


### SALMAGUNDI.

EDITED BY E. G. BETTY, D.D.S.

AN ANTHROPOLOGICAL CURIOSITY.—A double scull race.

IODOFORM is said to lose all its perfume if mixed with one-twelfth its weight of naphthalene.

TO DISGUISE ODOR OF IODOFORM.—The odor may be disguised by the addition of one minim of the ottar of roses to the ʒi of iodoform.

IODOL.—If mixed with wax it forms a good root filling, and with gutta-percha or osteo, supplies an excellent antiseptic stopping. V. A. L.

BREATHE through your nose; it was made for that purpose. Don't go around with your lower jaw hanging down like a bell-clapper. Sleep with your mouth closed.—*Med. Register*.

THE United States Government has paid vastly more money for investigating the diseases of hogs than those of human beings. Hogs, it seems, are "wealth," while men merely property owners.

WARTS.—Papayotiæ 12 grs. Water - - - ʒii

Borax - - 5 grs.

Paint over the parts twice a day, which will be found to cure the growth.

SORE-THROAT.—A gargle made of strong black tea and used cold night and morning, is now the fashionable preventative in London against falling a victim to sore throat during the cold winds of the spring.

A gargle of common salt ʒi Oss of water is an excellent preventative of sore throat and a nasal douche of the same is a most effective remedy for *chronic* coryza.

A PHYSICIAN says: "If a child does not thrive on fresh milk, boil it?" This is too severe. Why not spank it?—*Sinapore Review.*

---

THE Medical Examining Board of Virginia has examined thirty-three graduates in medicine for license to practice medicine in Virginia. Twenty-six of the applicants passed, and seven were rejected.

---

TEACHER: "Children, there is something within you that tells you when you have done wrong. What is it?" Small boy (gorged with green apples): "I know, sir. It's the colic."—*Detroit Free Press.*

---

THE Faculty of the University of Pennsylvania have banished cigarettes from the college grounds on the recommendation of Prof. J. Wm. White, who has charge of the physical education of the students.—*Sanitary Inspector.*

---

A RECENT writer in the *British Medical Journal* declares that excessive tea drinking is the cause of early decay of the teeth, possibly through so altering the normal secretions of the mouth as to permit the development of acids and micro-oranisms.

---

A FREEZING mixture which will reduce the temperature from ×35°F. to –23°F. may be made, according to O. Bachmann (*Rundschau. Prag.*), by mixing 112 parts each of concentrated sulphuric and nitric acids with 100 parts of snow.

---

BARFF'S BORO-GLYCERIDE.—One ounce in 12 ounces of distilled water forms a valuable lotion for all granulating wounds of the mouth after difficult extractions, and is useful in all inflammatory conditions of the oral mucous membrane.

---

DENTAL VARNISH.—A good quick-drying varnish for dental or other purposes is made as follows:

| | | |
|---|---|---|
| ℞ | Saturated solution of iodol in alcohol - - | 1 part. |
| | Hubbard's Negative Varnish - - - - | 6 " |

MAMMA: "Did you have a nice time at the party, Harry?" Harry: "Oh, yes, splendid! I had five kinds of cake. First I had some sponge-cake and chocolate-cake, and then I had jelly-cake and cream-cake, and then I had the stomach-cake."

---

A: "WHAT dentist made your teeth for you"? B: "These are my own teeth. No dentist made them." "You don't say so! How deceptive they are! Why, they look as nice as the best kind of false teeth. What a wonderful thing nature is."

---

POWDER FOR USE IN PYROSIS.—

| | | |
|---|---|---|
| ℞ | Pulv. phosphate of zinc - - | 10 parts. |
| | Calcined magnesia - - - | 3 " |
| | Pulv. Vanilla - - - - | 1 " |

ℨi in a wineglass full of water.

---

Dr. Emil B. Gardette, the President of the Board of Trustees of the Jefferson Medical College, died June 17, 1888, at his residence in Philadelphia, in the eighty-fifth year of his age. He was graduated from the Jefferson Medical College in 1838, and subsequently became well-known as a dentist.

---

TOOTHACHE A FATAL DISEASE.—In a back number of the *Times* (London) it says we must hereafter regard toothache as a fatal disease (?) The Behar paper contains the following announcement "Babu Benarasse Sal, one of the leading pleaders of the Judges' Court Sarum, died at Calcutta, of toothache on the 9th inst.

---

ABOUT seven years ago George W. Mitchell, of Palatka, Fla., falling against a cactus-plant, one of its thorns entered the calf of his leg. Several weeks later, the thorn was withdrawn from a swelling on his chin. This story is a welcome change from the old newspaper item about a girl running a needle into her thumb, which caused her no pain until six years subsequently, when it was removed from the ankle of her brother-in-law; but it is about as hard on a man's credulity.

A GENERAL antidote for poisons, according to the *Amer. Jour. of Pharmacy*, May 1888, may be made by mixing equal parts of calcined magnesia, wood charcoal, and hydrated oxide of iron, and is applicable in cases in which the poison is unknown. It should not, of course, supersede the stomach pump or other forms of emesis.

---

RIGG'S DISEASE. Sulphate of copper has been used with much advantage in the treatment of Rigg's Disease. The drug is best applied in powder with a strip of wood well under the gum and around the denuded fangs. After the application the mouth should be rinsed with an *alkaline* lotion. In severe cases successive daily treatment is necessary.

---

MAGISTRATE (to prisoner): "You say, Uncle 'Rastus, that you took the ham because you are out of work and your family is starving. And yet I understand that you have four dogs about the house."

Uncle 'Rastus: "Yes, sah, but I wuddent ask my family to eat dogs, yo' honah!"—*Boston Journal of Health.*

---

A REMEDY FOR NEURALGIA.—It is claimed that a few drops of the following: Eau de cologne, ether, chloroform, aa ℥iij, poured on a handkerchief previously wetted with cold water, and placed on the seat of a neuralgic pain, gives instantaneous relief. It is also very efficacious for nervous headache. A burning sensation is felt at first, but quickly disappears.—*Med. Record.*

---

MOTOR POWER.—Whilst some are using electro-motors for driving their engines, the majority of dentists are still content with foot power. In addition to the noise and increased vibration transmitted directly to the patient's head, the objection of failing batteries still detracts from the Electro-Motor Engine. A solution which can be strongly recommended is:

| | | | | | |
|---|---|---|---|---|---|
| R Chromic acid | - | 8 ozs. | Sulphuric acid | - | grs xvi |
| Water - - | - | 8 lbs. | | | |

Dissolve the chromic acid and add the sulphuric acid gradually while stirring.

An instantaneous light for taking photographs at night is obtained by burning a small quantity of a powder made of coarsely crushed sugar one part, magnesium powder one part, powdered chlorate of potassium two parts. The sugar must not be in too fine a powder or the mixture will burn with explosive violence. A mixture of equal parts of picric acid and potassium chlorate may be used, but is more dangerous.—*Rundschau. Prag.*

---

Chloride of Sodium has a wider range of use in medicine than many are aware of. As a remedy for anemic conditions it is recommended in a daily dose for an adult of 150 grains, in addition to what is ordinarily taken with the food. Bonchardat says that well salted meat diminishes the thirst of diabetic patients and lessens the excretion of sugar. Nothnagel recommend an epileptic patient to carry salt with him and swallow a quantity at the first sign of the *aura*. Rabow gives a half teaspoonful in migraine or sick headache—cures in half an hour. With some patients it is an infallible remedy. Salt is antidotal to nux vomica, curare, and to lead and silver salts. It should not be given for some hours after administering calomel. Salt counteracts the depressing effects of saline laxatives.—*Drug Topics*

---

The Care of the Nails.—Very few people know how to properly care for the nails. In cleaning them, a sharp knife ought never to be employed, but between the ends of the nails and the fingers the space should be filled with soap, and then removed by brushing with the so-called nail brush. Many improperly cut away that part of the flesh which grows over the nail from the bottom; but it should be simply pressed backward, and sufficiently to show the white part, considered by some to be a mark of beauty. If the flesh is adherent to the nail the operation may be facilitated by passing the sharp point of a knife underneath the fold of flesh and separating it from its attachments. With this done, in can be pushed back more readily. cissors should never be used to cut the nails; that should be done only with a sharp penknife-—*Boston Journal of Health.*

